# Unexpected difficult airway management and usefulness of high-flow nasal oxygenation for awake fiberoptic tracheal intubation: a case report

**DOI:** 10.1186/s40981-026-00850-y

**Published:** 2026-02-06

**Authors:** Takero Arai, Ryosuke Osawa, Takashi Asai

**Affiliations:** Department of Anesthesiology, Dokkyo Medical University Saitama Medical Center, 2-1- 50 Minami-Koshigaya, Koshigaya, Saitama 343- 8555 Japan

**Keywords:** High-flow nasal oxygenation, Fiberoptic intubation, Difficult airway

## Abstract

**Background:**

Unexpected difficult airway management, although rare, may occur after induction of anesthesia. In such cases, appropriate airway management is required to prevent life-threatening hypoxia.

**Case presentation:**

In a 44-year-old woman without predicted difficult airway, after induction of general anesthesia, repeated attempts at tracheal intubation using videolaryngoscopes had failed, and ventilation using both a facemask and a supraglottic airway became difficult; the patient was awakened and the surgery cancelled. One week later, awake nasotracheal intubation using a flexible bronchoscope was attempted under high-flow nasal oxygenation. A clear view of the glottis was obtained by bronchoscopy and the trachea was intubated successfully without hypoxia.

**Conclusions:**

In patients with unexpected difficult airway management, appropriate airway management (including awakening the patient) is required to prevent life-threatening hypoxia. High-flow nasal oxygenation would be useful for awake fiberoptic intubation, by providing a clear view of the glottis and by preventing hypoxia.

## Background

The usefulness of videolaryngoscopes is now well recognized, and the recent guidelines on airway management recommend using a videolaryngoscope as the first line to facilitate tracheal intubation whenever possible [1, 2]. Nevertheless in rare cases, tracheal intubation using a videolaryngoscope may be difficult or impossible [3, 4], and if such difficulties occur in anesthetized patients, appropriate airway management, including awakening the patient from general anesthesia, is required [1, 2, 5].

Tracheal intubation using a flexible “fiberoptic” bronchoscope is regarded as the gold standard technique for managing predicted difficult airways [1, 6–8]. However, sedation or general anesthesia may frequently cause upper airway obstruction, making it difficult to locate the glottis through a flexible bronchoscope [6, 7]. In addition, if hypoxia occurred, attempt of intubation should be interrupted, and manual ventilation through a facemask may be required.

High-flow nasal oxygenation (HFNO) [9], which delivers heated and humidified oxygen at flow rates up to 70 L/min, has increasingly been used during induction of anesthesia and during procedures under sedation [9–11]. There have also reports of HFNO being used during awake fiberoptic intubation in patients with predicted difficult airways [12–14], but not in patients with history of failed intubation. HFNO may not only delay the occurrence of hypoxia but also help in maintaining a patent airway and improving the view of the glottis, by generating a low level of continuous positive airway pressure [11, 15].

We report a successful use of HFNO during awake fiberoptic intubation in a patient in whom tracheal intubation after induction of anesthesia had failed.

## Case presentation

Written informed consent was obtained from the patient for publication of this case report.

A 44-year-old woman (160 cm, 62 kg) was scheduled to undergo total abdominal hysterectomy with bilateral salpingo-oophorectomy and omentectomy for suspected ovarian malignancy. The patient had no significant medical history (including no sleep apnea syndrome), and was classified as American Society of Anesthesiologists (ASA) physical status 1.

**Fig. 1:**
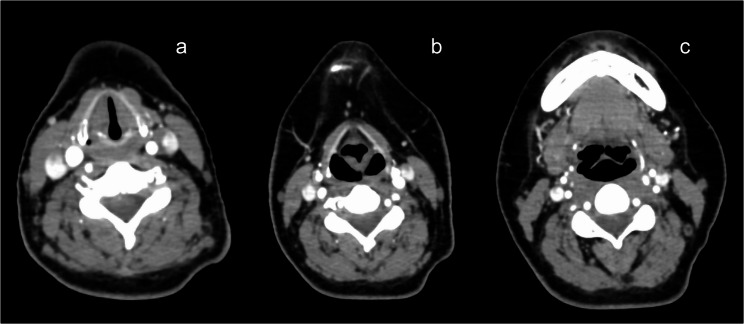
Preoperative computed tomography images of the neck (**a** glottic level, **b** arytenoid level; **c** epiglottic level): no apparent anatomical abnormalities of the larynx can be found

Preoperative evaluation indicated no features suggestive of difficult airway: mouth opening was greater than three finger breadths, neck movement was not restricted, and no upper airway obstruction was suspected. Both trainee anesthetist and senior anesthetist checked the chest Roentgenogram and computed tomography (CT, which was taken for screening of lymph node metastasis) (Fig. 1) and found no apparent anatomical abnormalities which would make airway management difficult.

**Fig. 2:**
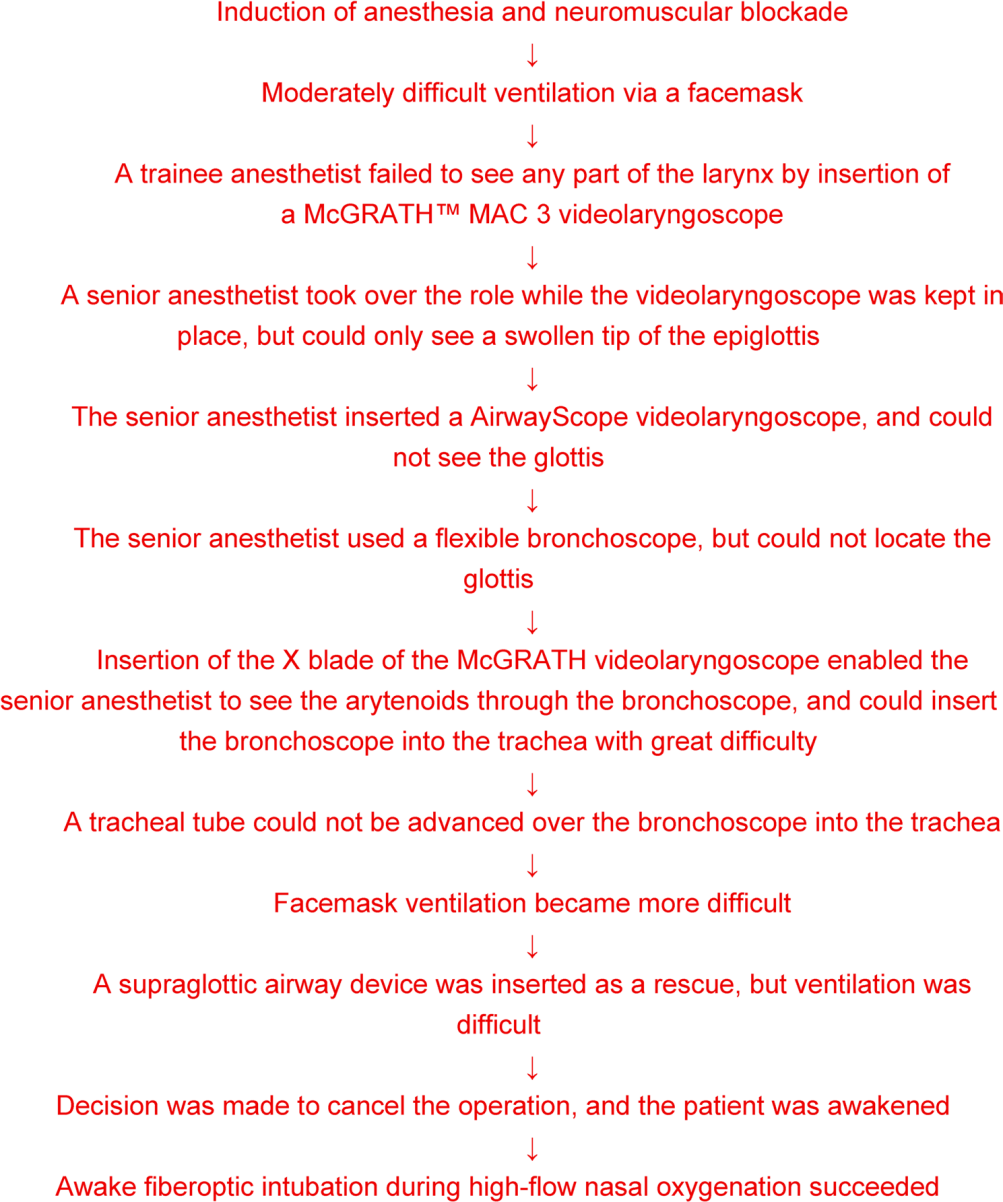
Sequence of airway management in a patient with unpredicted difficult airway

In an operating room, after insertion of an epidural catheter, general anesthesia was induced with propofol 150 mg, fentanyl 100 µg, and remifentanil 0.2 µg/kg/min, and neuromuscular blockade was achieved with rocuronium 50 mg. Sevoflurane 2% was started, and manual ventilation through a facemask was attempted, but it was moderately difficult (Fig. 2). With thrusting the jaw forward, it became easier. After adequate neuromuscular blockade was confirmed using a neuromuscular monitor, a trainee anesthetist inserted the McGRATH™ MAC 3 videolaryngoscope (Medtronic, Massachusetts, USA) to the oral cavity, but could not see any part of the larynx. A senior anesthetist who was confirming the view on the videolaryngoscope monitor immediately took over the role by instructing the trainee to keep the blade in place. There was difficulty in advancing the blade beyond the angle between the oral axis and the upper pharyngeal axis, and only a somewhat swollen tip of the epiglottis could be seen, but not the glottis. Insertion of the AirwayScope videolaryngoscope also failed to see the glottis.

As the third attempt, a flexible bronchoscope (which was passed through a 6.0-mm internal diameter reinforced tracheal tube (Shiley™, Idaho, USA)) was introduced into the oral cavity, but because of a narrowed pharyngeal upper pharynx, it was not possible to advance the tip of the bronchoscope to locate either the epiglottis or the glottis, even with the aid of bilateral jaw thrusting. Insertion of the X blade of the McGRATH videolaryngoscope did not improve the view of the glottis, but by inserting a flexible bronchoscope with the X blade left in place, it became possible to see the arytenoids. The bronchoscope could be inserted to the trachea with great difficulty because of a swollen and deformed epiglottis covering the glottic opening. Nevertheless, a tracheal tube could not be advanced over the bronchoscope to the trachea, possibly because the tip of the tracheal tube kept impacting to the epiglottis or to the arytenoids. Mask ventilation became more difficult (Fig. 2).

As a rescue device, a size 4 supraglottic airway device (I-gel™: Intersurgical, Berkshire, UK) was inserted, and it was possible to ventilate the lungs manually, but there was still difficulty in inflating the lungs. Arterial hemoglobin oxygen saturation was remaining high (99–100%). Because operation would take several hours, and because there was a risk of laryngeal edema due to repeated attempts at tracheal intubation, we judged it necessary to intubate the trachea. By inserting the bronchoscope to the breathing tube of the i-gel, it became apparent that the epiglottis was obstructing the distal aperture of the i-gel, making it impossible to intubate the tracheal through the i-gel and making it impossible to assess if the glottis was edematous.

Because of repeated failed intubation, and because surgeons told us that surgery could be postponed, the decision was made to wake the patient up. Sugammadex 200 mg was injected intravenously to reverse neuromuscular blockade. After the train-of-four on the neuromuscular monitor had increased greater than 90, the patient was awakened without complications. The procedure was postponed (Fig. 2). No airway complications occurred afterwards.

A week later, operation was planned. Another anesthetists re-assessed the patient’s airway as well as chest Roentgenogram and CT images, and did not find any anatomical deformities indicative of difficult airway.

In the operating room, routine monitors including a non-invasive blood pressure cuff, an electrocardiogram, a pulse oximeter, and a bispectral index (BIS) monitor, were applied. HFNO was started using a medium-sized nasal cannula (Optiflow™, Fisher & Paykel Healthcare, New Zealand), with 100% oxygen at a flow rate of 40 L/min. Sedation was initiated using dexmedetomidine at 4 µg/kg/h. After 10 min, fentanyl 25 µg was administered intravenously, and dexmedetomidine was reduced to 0.5 µg/kg/h. Topical anesthesia to the nasal cavity was applied using swabs soaked in 4% lidocaine with epinephrine. Additionally, 2% lidocaine syrup was administered orally, and the nasal cavity was disinfected with povidone-iodine.

Because the BIS remained above 90 and the patient was still awake, the dexmedetomidine was increased to 0.7 µg/kg/h, and fentanyl 25 µg was administered. HFNO was then increased to 70 L/min. A flexible bronchoscope was inserted through the left nostril alongside the nasal cannula. A clear view of the glottis was easily obtained, and 0.5 mL of 4% lidocaine was sprayed through the working channel of the bronchoscope.

Fentanyl 25 µg was administered, and the patient was instructed to take a deep breath. A 6.0-mm internal diameter reinforced tracheal tube was then easily advanced over the bronchoscope into the trachea. The bronchoscope was withdrawn, the tube was connected to the anesthesia breathing system, and the correct tracheal intubation was confirmed by the presence of the end-tidal carbon dioxide waveforms. General anesthesia was then induced with propofol 100 mg, and neuromuscular blockade was achieved with rocuronium 50 mg. SpO₂ remained 99% or 100% throughout the procedure. The surgery proceeded without complications, and at the end of the procedure, the patient’s trachea was extubated smoothly without complications.

**Fig. 3:**
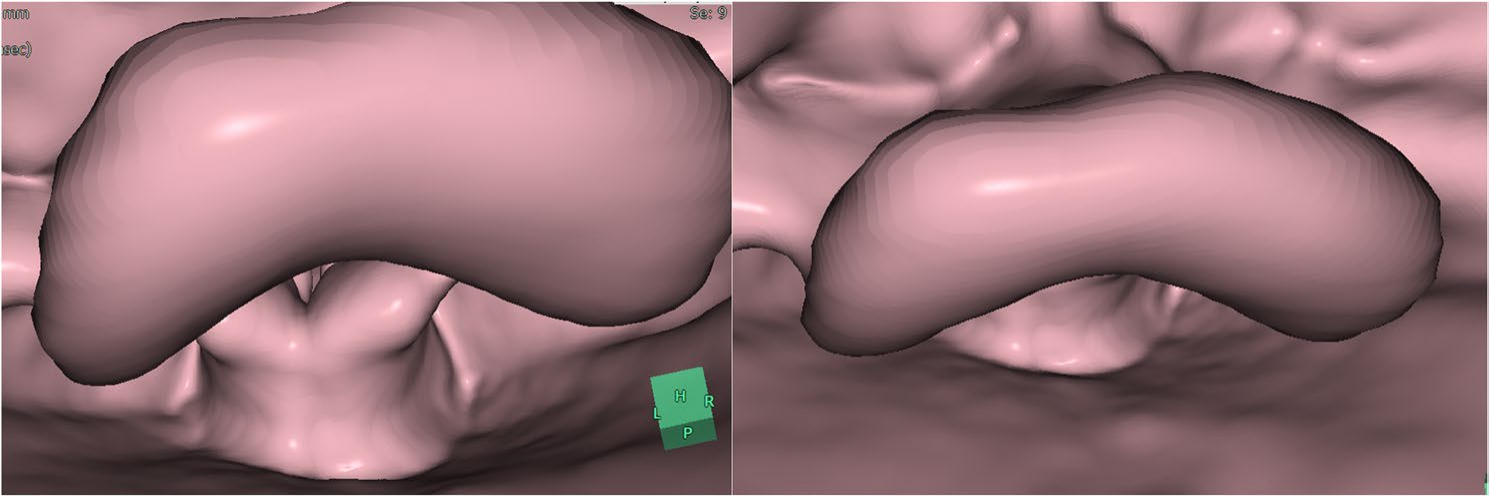
Three-dimensional images of the larynx constructed from the preoperative computed tomography images of the neck: a swollen and deformed epiglottis was obstructing the airway passage by covering the glottis

Postoperatively, we closely examined the preoperative CT images to find out the cause of difficult airway management. By constructing a 3-dimensional images, we have found that a swollen and deformed epiglottis was covering the glottis (Fig. 3), and have concluded that a swollen and deformed epiglottis would have been the major cause of difficulty in manual ventilation (via a facemask and via a supraglottic airway) and in viewing the glottis (at laryngoscopy and at bronchoscopy), and for obstructing the distal aperture of the supraglottic airway.

## Discussion

We experienced a case of unpredicted difficult intubation, where attempts at tracheal intubation using videolaryngoscopes and flexible bronchoscopy failed after induction of general anesthesia.

The exact cause of difficult tracheal intubation in our case is not clear, but the main cause would have been a swollen and deformed epiglottis. We could not find out this anatomical abnormality from physical examinations, x-ray images and the CT images taken before induction of anesthesia (Fig. 1), but could only find out after cancellation of surgery, by re-reviewing the CT images and by constructing 3-dimensional images of the larynx from the preoperative CT images (Fig. 3). Therefore, we recommend that if the patient has a history of difficult airway management, 3-dimensional image constructed from the CT images should also be assessed before carrying out airway management.

These CT images were taken while the patient was fully awake. It is likely that, after induction of general anesthesia and neuromuscular blockade, this swollen epiglottis was collapsed toward the posterior pharyngeal wall and toward the glottis, and interfered with both laryngoscopy and manual ventilation.

The guidelines for airway management recommend that, when tracheal intubation is difficult, repeated attempts at laryngoscopy should be avoided, because repeated attempts increase the risk of airway obstruction with hypoxia [1, 2, 5]. If facemask ventilation has become difficult, a supraglottic airway may be inserted as a “rescue” device, and if ventilation through a supraglottic airway has also failed, either a front-of-neck access (such as cricothyrotomy) should be established, or the patient should be awakened [1, 2, 5]. In our case, we attempted to secure the airway by complying with the recommendations of these guidelines, and experienced increasing difficulty in ventilation through a facemask and through a supraglottic airway. Because ventilation had become more difficult and because surgeons stated that surgery could be postponed, we decided to wake the patient up (by reversing neuromuscular blockade and by terminating administration of anesthetics), instead of making a front-of-neck access.

The exact cause of increasing difficulty in ventilation after repeated attempts at laryngoscopy would frequently not be clear, because it would be difficult or impossible to see the larynx, as in our case, to confirm if the larynx has become edematous. Nevertheless, we should assume that increasing difficulty in ventilation is caused by narrowing the upper airway by edema, and thus we should avoid repeated attempts at laryngoscopy.

Awake fiberoptic intubation is indicated in such cases, and the guidelines for awake tracheal intubation recommend oxygen supplementation [8]. According to the guidelines [8], high-flow nasal oxygenation is associated with a lower incidence of hypoxia during awake tracheal intubation, compared with low-flow oxygen delivery.

HFNO may not only maintain oxygenation, but also may improve the view of the glottis during attempt at fiberoptic intubation, by generating a continuous positive airway pressure and widening the pharyngeal space [7]. In our patient, fiberoptic intubation had failed when the patient was under general anesthesia, but both insertion of a flexible bronchoscope to the trachea and tracheal intubation were easy when the patient was sedated and high-flow nasal oxygenation was being used. It is likely that high-flow nasal oxygenation widened the oropharyngeal cavity and facilitated location of the glottis through the flexible bronchoscope.

It has been shown that the level of the continuous positive airway pressure produced by HFNO increases as oxygen flow rate increases [15]. Therefore, it is advantageous to increase the oxygen flow rate to the maximum of 70 L/min, to obtain a clearer view of the glottis, by widening the pharyngeal space. Nevertheless, patients may have unpleasant feeling when 70 L/min is provided. Therefore, a lower flow, such as 30–40 L/min, may be suitable for awake or lightly sedated patients [9, 10]. This is the reason why we provided 40 L/min when the patient was awake, and increased to 70 L/min after the patient was sufficiently deeply sedated.

Although the presence of the nasal cannula for high-flow oxygenation may theoretically interfere with insertion of a flexible bronchoscope, with careful insertion, we did not have difficulty in maneuvering the bronchoscope without dislodging the nasal cannula. We used a medium-sized nasal cannula for high-flow oxygenation, but if there is difficulty in insertion of a flexible bronchoscope, a narrower nasal cannula may be used.

In conclusion, high-flow nasal oxygenation would be useful for awake fiberoptic intubation in patients with difficult airways, both by preventing of hypoxia and by providing a clear view of the glottis.

## Data Availability

Not applicable.
